# PD-L1 Immuno-PET Reveals Systemic Effects of Localized Oncolytic Virotherapy in a Mouse Model of Head and Neck Cancer

**DOI:** 10.2967/jnumed.125.270922

**Published:** 2026-06

**Authors:** Julia Höbart, Chiara Da Pieve, Florian Raes, Damian Borys, Justyna Mączyńska, Gitanjali Sharma, Alan A. Melcher, Kevin J. Harrington, Gabriela Kramer-Marek

**Affiliations:** 1 Division of Radiotherapy and Imaging, Institute of Cancer Research, London, United Kingdom;; 2 Department of Systems Biology and Engineering, Silesian University of Technology, Gliwice, Poland; and; 3 Department of Nuclear Medicine and Endocrine Oncology, Maria Skłodowska‐Curie National Research Institute of Oncology, Gliwice Branch, Gliwice, Poland

**Keywords:** head and neck cancer, PD-L1, oncolytic virus, ^89^Zr, immuno-PET

## Abstract

Immune checkpoint inhibitors can trigger antitumor immunity, yet most patients, including those with head and neck cancer, show limited benefit because of insufficient immune activation at the tumor site. Oncolytic virotherapy (OV) may overcome this by priming tumors at the injection site for a stronger response to immune checkpoint inhibitors. However, there are limited data on how OV influences systemic immune responses in uninjected tumors and secondary lymphoid organs (spleen, lymph nodes), largely because of the difficulty of sampling these tissues. We investigated whether immuno-PET can noninvasively track systemic and intratumoral changes in programmed death ligand 1 (PD-L1) expression after intratumoral OV administration in a syngeneic mouse model of oral carcinoma. **Methods:** ^89^Zr-DFO-PD-L1_mAb_ was injected into murine oral cancer (MOC) models, including MOC1, MOC2, and MOC2(*PD-L1*) tumor–bearing mice. Whole-body PET/CT static scans were performed 48 h after injection. On the basis of in vitro OV sensitivity, the MOC1 model was selected to study the effects of OV on PD-L1 expression after a single intratumoral dose of RP1, a proprietary strain of oncolytic herpes simplex virus. Immuno-PET scans with concomitant biodistribution studies were performed on days 3 and 7 after OV administration. Radiomics features were extracted from immuno-PET scans, and immunohistochemistry was performed to confirm PD-L1 expression levels. Intratumoral cytokines and CD8 T-cell infiltration were also assessed. **Results:** RP1 injection significantly increased ^89^Zr-DFO-PD-L1_mAb_ uptake in spleens and tumor-draining lymph nodes, as observed in the immuno-PET images acquired 3 d after treatment. By day 7, uptake levels in these organs returned to pretreatment levels. In contrast, no transient increase in radioconjugate uptake was observed in tumors treated with RP1 compared with controls. The levels of intratumoral and intrasplenic ^89^Zr-DFO-PD-L1_mAb_ uptake were consistent with PD-L1 immunohistochemistry performed on representative sections. Radiomics analysis of tumors and spleens revealed several features occurring on day 3 after administration of OV. Elevated levels of intratumoral type I interferon, followed by CD8 T-cell infiltration, indicated local immune stimulation. **Conclusion:** The present work highlights the potential of anti–PD-L1 immuno-PET to noninvasively assess spatiotemporal changes in PD-L1 expression on a whole-body scale. This method can help guide and optimize OV, a task that would be challenging to achieve with a single biopsy alone.

## Visual Abstract

**Figure:**
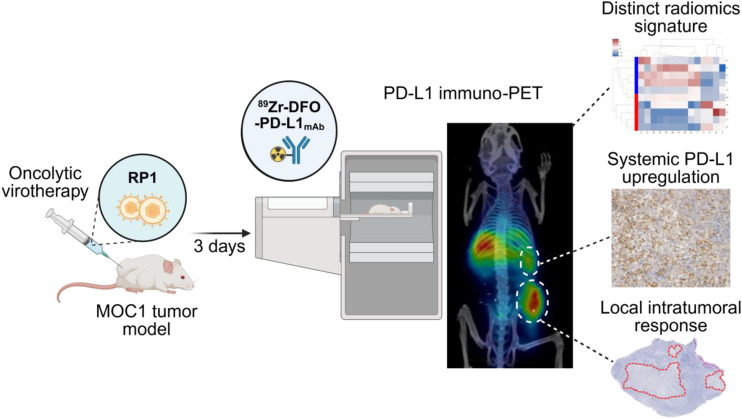

The management of locally advanced head and neck squamous cell carcinoma (HNSCC) typically involves multimodal approaches combining surgery, radiotherapy, and chemotherapy. Despite these aggressive interventions, clinical outcomes remain suboptimal, with more than half of patients with HNSCC experiencing disease recurrence or progression within 5 y of diagnosis (1). Additionally, because of the radical nature of conventional treatments and the complex anatomy of the head and neck area, most patients are left with permanent therapy-associated side effects. Therefore, there is an urgent clinical need to explore more effective and tolerable treatment strategies to improve overall therapy outcomes in these patients. The advent of immune checkpoint inhibitors (ICIs) targeting either the programmed death receptor 1 (PD-1) or its ligand (PD-L1) has revolutionized the therapeutic landscape across various cancer types. Both anti–PD-1 and anti–PD-L1 agents are designed to interfere with an important T-cell inhibitory axis, thereby reinvigorating an immune response against cancer (2). This immunotherapeutic strategy has shown promising clinical outcomes in multiple tumor entities and has led to the approval of 2 anti–PD-1 monoclonal antibody (mAb)–based treatments of cisplatin-refractory, metastatic, or unresectable HNSCC (3,4). However, not all patients with HNSCC benefit from anti–PD-1 blockade, most likely because of the presence of a highly immunosuppressive tumor microenvironment (TME) (5,6). Consequently, strategies aimed at converting the highly immunosuppressive milieu into an immune-responsive state, before ICI administration, may improve therapeutic outcomes in this patient subset. One strategy to achieve this relies on oncolytic virotherapy (OV).

OVs are a promising class of cancer immunotherapies that can selectively infect and destroy cancer cells without harming healthy tissue (7). Importantly, OVs have the capability to attract immune cells to the tumor as the main site of infection, thereby holding great promise for synergistic effects with anti–PD-1 and anti–PD-L1 blockers (8). Additionally, OVs can trigger local interferon (IFN) responses, resulting in elevated PD-L1 expression, which boosts the effectiveness of ICIs targeting PD-1 and PD-L1 (9). Indeed, OV and ICI combinations have been effective across different types of cancer in preclinical and clinical settings. Recent examples include the combination treatment of oncolytic *Vaccinia* virus with anti–PD-1 and anti–PD-L1 mAbs in murine models of colon and ovarian carcinoma (10,11). Likewise, studies using a bilateral CT26 tumor model showed an upregulation of PD-L1 in both the OV-injected and noninjected lesions, demonstrating that intratumoral OV administration can elicit systemic immunomodulatory effects (11). Furthermore, upregulation of PD-L1 after OV administration has been observed in several other cancer models, including myeloma, acute leukemia, and glioma (12–14). Previous clinical studies have also demonstrated increased levels of PD-L1 in glioma and melanoma brain metastases and in a subset of patients with pancreatic cancer after intravenous reovirus injection (9,15). However, the only clinically approved OV to date is the oncolytic herpes simplex virus (oHSV) talimogene laherparepvec (Imlygic; Amgen) (16). Furthermore, the effects of clinically relevant oHSVs in HNSCC, with a focus on the systemic expression of the immune checkpoint PD-L1, remain poorly understood. Therefore, we used immuno-PET to assess changes in PD-L1 expression after administration of RP1 (Replimune), a proprietary strain of oHSV (17), in a mouse model of HNSCC.

PD-L1 levels on tumor and immune cells shift constantly, both over time and between regions of the same tumor, particularly after exposure to IFNs and other cytokines released in response to OV (18). Whereas conventional diagnostic tools, such as biopsies, can capture only snapshots of the tumor, immuno-PET can visualize dynamic changes across all sites and can be valuable to noninvasively map the PD-L1 expression throughout the body in real time (19,20). Additionally, previous preclinical (21–23) and clinical work (19,24) has shown that ^89^Zr-labeled anti–PD-L1 mAbs reliably depict PD-L1 modulation in vivo. Accordingly, we tested whether ^89^Zr-DFO-PD-L1_mAb_–based imaging could reveal both systemic and intratumoral PD-L1 upregulation after treatment with the oHSV RP1 in a syngeneic HNSCC model.

## MATERIALS AND METHODS

### Cell Culture

Murine oral carcinoma (MOC) cell lines with different sensitivities to OV and low PD-L1 expression levels (MOC1, MOC2) were provided by Ravindra Uppaluri from the Dana-Farber Cancer Institute. The MOC2(*PD-L1*) cell line was generated in our laboratory to overexpress PD-L1 by transducing MOC2 cells with a lentiviral vector carrying the murine *CD274* gene tagged with a green fluorescent protein (GFP) reporter (MR 203953L2; OriGene Technologies). The GFP-positive cell population was isolated using a MoFlo Astrios cell sorter (Beckman Coulter). All cell lines were cultured in Dulbecco’s Modified Eagle Medium supplemented with 10% heat-inactivated fetal bovine serum (both Thermo Fisher Scientific) at 37 °C in a humidified chamber containing 5% CO_2_. *Mycoplasma* negativity was routinely confirmed via polymerase chain reaction (Surrey Diagnostics).

### Flow Cytometry

MOC1, MOC2, and MOC2(*PD-L1*) cells (3–5 × 10^5^) were resuspended in phosphate-buffered saline (PBS) with 2% fetal bovine serum and incubated with phycoerythrin-labeled anti–PD-L1 or IgG2b control antibody (1:200; BioLegend) and Draq7 viability dye (1:50 dilution factor; BioStatus) for 30 min at 4 °C. Data were acquired on an LSRII flow cytometer and analyzed using FlowJo software (both BD Biosciences).

### Confocal Microscopy

For confocal microscopy imaging, 3–4 × 10^5^ cells were seeded into a Nunc glass bottom petri dish (Thermo Fisher Scientific) and then stained and imaged as described in the supplemental materials, available at http://jnm.snmjournals.org (25–29).

### Immunoblotting

Western blotting was performed as described previously (30). Detailed methods are provided in the supplemental materials.

### Preparation of ^89^Zr-DFO-PD-L1_mAb_

The conjugation of *p*-isothiocyanatobenzyl-deferoxamine (CheMatech) to PD-L1_mAb_ (antimouse PD-L1 mAb, clone 10F.9G2; BioXCell) and the preparation and in vitro serum stability of ^89^Zr-DFO-PD-L1_mAb_ are described in the supplemental materials.

### ^89^Zr-DFO-PD-L1_mAb_ In Vitro Immunoreactivity, Affinity, and Binding Specificity

The immunoreactive fraction, the dissociation constant, and the binding specificity were determined as previously described (31,32). Experimental details are listed in the supplemental materials.

### Mouse Models

All animal studies were performed in accordance with the license issued under the U.K. Animals (Scientific Procedures) Act 1986, the U.K. National Cancer Research Institute Guidelines for Animal Welfare in Cancer Research (33), and the ARRIVE guidelines for reporting animal research. All experiments were conducted under the project license P5B619C3D, approved by the U.K. Home Office, and by the local ethical review committee. Female C57BL/6J mice (Charles River U.K.) aged 7–9 wk were subcutaneously injected on the left flank with either MOC1, MOC2, or MOC2(*PD-L1*) cells (1 × 10^6^ in 100 μL of PBS). Tumor volumes were assessed via caliper measurements using the following formula: *V* = 1/2 × length × width^2^. Humane endpoints were specified with a tumor diameter of greater than 15 mm or tumor ulcerations greater than 5 mm in diameter.

### PET Imaging and Biodistribution Studies

^89^Zr-DFO-PD-L1_mAb_ (2 MBq, 14–165 μg) was injected via the tail vein into mice bearing MOC1, MOC2, and MOC2(*PD-L1*) tumors (93 ± 33 mm^3^). Whole-body PET static scans were performed 48 h after injection using an Albira PET/SPECT/CT scanner (Bruker) for 15 min, followed by a 10-min CT scan under continuous isoflurane anesthesia (approximately 2% v/v in O_2_). The detailed reconstruction and analysis protocols are provided in the supplemental materials. Biodistribution studies were performed after scanning. Major organs were collected and weighed, and their radioactivity measured on a γ-counter (Wizard^2^ 2480; PerkinElmer).

### Ex Vivo Histopathology

Formalin-fixed paraffin-embedded tissues were cut into sections that were approximately 4-µm thick and mounted onto microscope slides. Detailed staining procedures and analysis methods are described in the supplemental materials.

### oHSV Variants

Two oHSV variants were used: RP1-16 (hereinafter referred to as RP1), expressing a murine granulocyte-macrophage colony-stimulating factor (GM-CSF) and a fusogenic glycoprotein gibbon ape leukemia virus, and RP1-15, expressing GFP instead of murine GM-CSF (17). Both viruses were produced and provided by Replimune.

### In Vitro Infectious and Cytopathic Capacity of RP1

The infectious ability and the cytopathic capacity of RP1 were tested in MOC1 and MOC2 cells in vitro. Detailed procedures are outlined in the supplemental materials.

### PD-L1 Assessment by PET/CT After OV

Mice bearing MOC1 tumors (131 ± 33 mm^3^) were injected intratumorally with a single dose of RP1 (1 × 10^6^ plaque-forming units [PFU] in PBS, 10 μL) or vehicle control (PBS, 10 μL) and intravenously injected with the radioconjugate (2 MBq, 110 μg). They underwent PET imaging on 3 or 7 d after therapy. A more detailed description of the procedure is provided in the supplemental materials.

### Radiomics

PET data from the tumors and spleens of MOC1 tumor–bearing mice were analyzed for radiomics features after treatment with RP1 or PBS, detailed in the supplemental materials. Briefly, all PET images were preprocessed and converted to NRRD format. Images were normalized to a maximum intensity value. Tumors were autosegmented using a 41% threshold of maximum voxel intensity, whereas spleens were manually segmented with itk-SNAP. In total, 100 radiomics features, including shape, first-order, and texture features, were extracted using PyRadiomics. Features were selected using the Mann–Whitney *U* test, and hierarchical clustering was performed for visualization.

### Ex Vivo Cytokine Analysis

Cytokine analysis was conducted on tumor samples collected 3 d after treatment with RP1 or PBS, as detailed in the supplemental materials.

### Statistical Analysis

All in vitro experiments were performed at least twice, with 2–3 replicates, except for the Lindmo assay, which was performed once. Statistical analyses were performed using Prism version 8 (GraphPad Software), unless otherwise specified.

## RESULTS

### PD-L1 Expression in MOC1, MOC2, and MOC2(*PD-L1*) Cells

**FIGURE 1.**
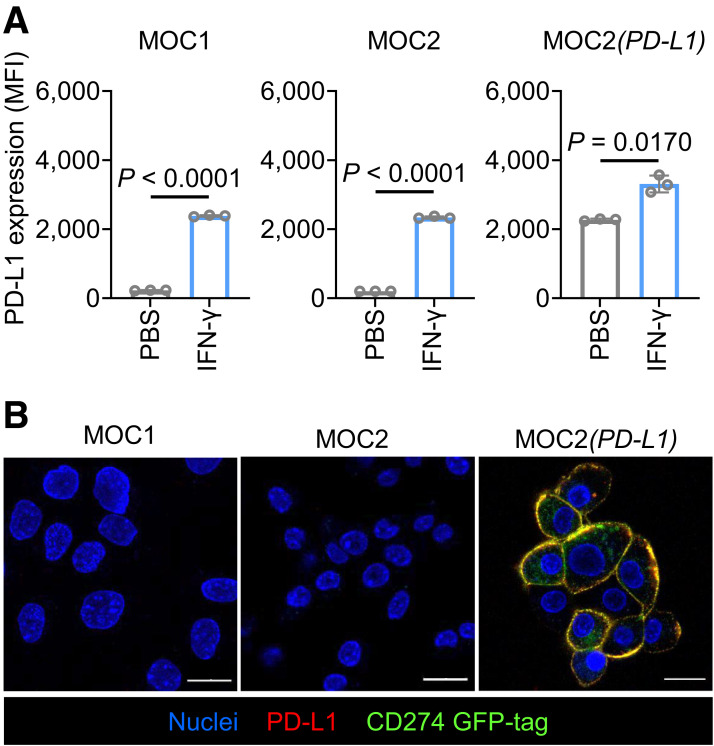
(A) Bar charts showing PD-L1 expression via flow cytometry. (B) Confocal microscopy images of MOC1, MOC2, and MOC2(*PD-L1*) cells merged from 3 channels (Supplemental Fig. 1A). Scale = 20 µm. MFI = median fluorescence intensity.

PD-L1 expression in MOC1, MOC2, and MOC2(*PD-L1*) cell lines was assessed by flow cytometry and confocal microscopy (Figs. 1A and 1B; Supplemental Fig. 1A). Baseline PD-L1 expression levels were low in MOC1 and MOC2 cells compared with the MOC2(*PD-L1*) cells. Incubation of the cells with IFN-γ resulted in a significant increase in PD-L1 expression, as confirmed by flow cytometry (Fig. 1A). This approach was used to mimic cytokine-mediated PD-L1 expression changes that frequently occur in tumors in vivo. The polyclonal MOC2(*PD-L1*) cell line was used as a positive control to evaluate the specificity of the ^89^Zr-DFO-PD-L1_mAb_ radioconjugate in vitro and in vivo.

### Preparation, In Vitro Stability, and Binding of ^89^Zr-DFO-PD-L1_mAb_

**FIGURE 2.**
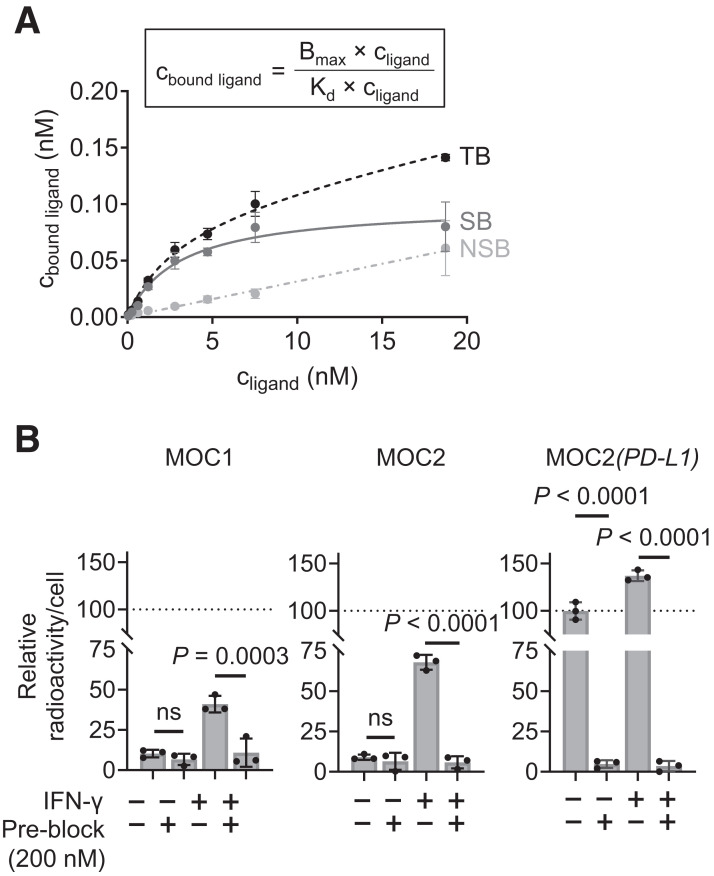
(A) Saturation binding assay of ^89^Zr-DFO-PD-L1_mAb_ on MOC2(*PD-L1*) cells. Curves for total bound (TB), specifically bound (SB), and nonspecifically bound (NSB) fractions fitted using given equation. (B) Specificity of binding assay of ^89^Zr-DFO-PD-L1_mAb_ (2 nM; 1 h) on cancer cell lines pretreated with IFN-γ (20 ng/mL; 24 h) or PBS. Blocking with 100-fold molar excess of unlabeled PD-L1_mAb_. Data were normalized to nontreated MOC2(*PD-L1*) cell–associated radioactivity. Significance determined using 2-way ANOVA with Bonferroni adjustment. C_bound_ = bound ligand concentration; C_ligand_ = ligand concentration; B_max_ = maximum specific binding; *K*_d_ = dissociation constant; ns = not significant.

^89^Zr-DFO-PD-L1_mAb_ was produced with a radiochemical yield of 45%–67.5%, an apparent specific activity of 0.11–0.16 MBq/µg (apparent molar activity, 16.5–23.9 MBq/nmol), and a radiochemical purity exceeding 98%. Stability studies demonstrated negligible demetalation of the radioconjugate (7.3%) in mouse serum over 7 d (Supplemental Fig. 2A). The immunoreactive fraction of ^89^Zr-DFO-PD-L1_mAb_ exceeded 80%, confirming that bioconjugation and radiolabeling had minimal impact on epitope binding (Supplemental Figs. 2B and 2C). Additionally, the dissociation constant calculated from a cell-based saturation assay using MOC2(*PD-L1*) cells was 2.41 ± 0.75 nM (Fig. 2A). The measured binding specificity of the radioconjugate matched PD-L1 expression levels determined by flow cytometry on MOC1, MOC2, and MOC2(*PD-L1*) cells (Figs. 1A and 2B). Moreover, cell-associated radioactivity was significantly higher in the cells stimulated with IFN-γ and decreased to baseline when PD-L1 was blocked by excess nonradiolabeled PD-L1_mAb_, confirming high target specificity of ^89^Zr-DFO-PD-L1_mAb_ (Fig. 2B).

### PET Imaging and Biodistribution Studies

A titration study with increasing quantities of PD-L1_mAb_ coinjected with ^89^Zr-DFO-PD-L1_mAb_ (2 MBq; total antibody protein doses of 60, 110, and 165 µg) was performed in mice bearing MOC2 (low PD-L1 expression) and MOC2(*PD-L1*) (high PD-L1 expression) tumors to determine the optimal antibody dose for PD-L1 imaging (Supplemental Fig. 3A; *n* = 3–6 mice per group). Biodistribution studies conducted 48 h after injection showed considerable changes in radioconjugate uptake corresponding to the PD-L1_mAb_ dose, primarily in the tumor, liver, and spleen (Supplemental Fig. 3B). High uptake in the liver indicated hepatobiliary clearance of ^89^Zr-DFO-PD-L1_mAb_. Radioactivity in the shoulders and knee joints was caused by ^89^Zr demetalation and hydroxyapatite affinity (34). The greatest difference in ^89^Zr-DFO-PD-L1_mAb_ uptake between MOC2 and MOC2(*PD-L1*) tumors was seen when a 110-µg protein dose was injected (21.1 ± 5.7 %ID/g vs. 32.7 ± 4.0 %ID/g, respectively) (Supplemental Fig. 3A). Consequently, this protein dose was applied in all further experiments.

**FIGURE 3.**
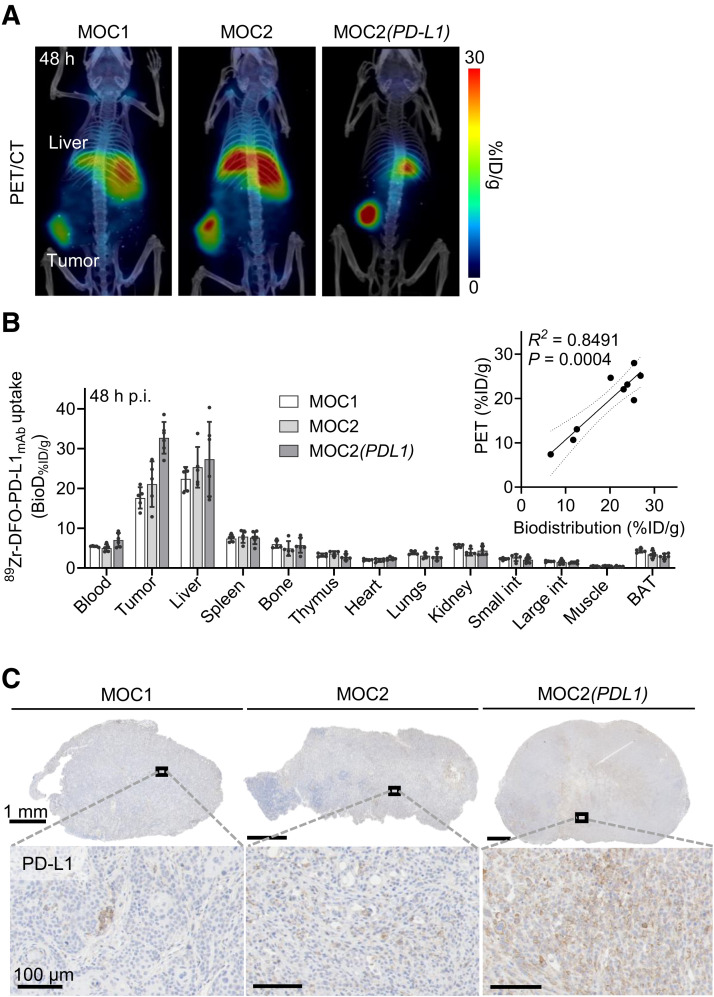
(A) Coregistered PET/CT scans of MOC1, MOC2, and MOC2(*PD-L1*) tumor–bearing mice 48 h after ^89^Zr-DFO-PD-L1_mAb_ injection (PET, coronal slice; CT, 3-dimensional maximum intensity projection). (B) ^89^Zr-DFO-PD-L1_mAb_ biodistribution profile of MOC1, MOC2, and MOC2(*PD-L1*) tumor–bearing mice 48 h after injection (110 μg; adjusted specific activity = 0.018 MBq/μg). Data pooled from 3 studies. Inset graph shows correlation of PET quantification and biodistribution (BioD). Linear regression fit with 95% CI (dashed lines). (C) PD-L1 immunohistochemistry of tumor sections. BAT = brown adipose tissue; p.i. = postinjection.

Next, the ability of ^89^Zr-DFO-PD-L1_mAb_ to image PD-L1 was compared in the 3 syngeneic models (MOC1, MOC2, and MOC2(*PD-L1*); *n* = 5–6 mice) at 48 h after injection. The radioconjugate clearly visualized the tumors, and uptake reflected the PD-L1 expression level in all models (Figs. 3A and 3C; Supplemental Fig. 1B). Image-based quantification data were corroborated by the biodistribution results (Fig. 3B), showing the lowest uptake in MOC1 tumors (17.6 ± 2.7 %ID/g), slightly more uptake in MOC2 (21.1 ± 5.7 %ID/g) tumors, and the highest uptake in MOC2(*PD-L1*) tumors (32.7 ± 4.0 %ID/g) (Supplemental Table 1). Pearson correlation analysis of a representative set of data from MOC2 tumors showed a strong correlation between PET and biodistribution-based quantification data (Fig. 3B). Tumor PD-L1 expression was also confirmed by immunohistochemistry (Fig. 3C). Anti-CD31 immunohistochemistry confirmed vascular endothelial cells in tumors and showed slightly higher vessel density in MOC2(*PD-L1*) tumors (Supplemental Figs. 4A and 4B).

### Capability of RP1 to Infect and Lyse MOC1 and MOC2 Cells In Vitro

**FIGURE 4.**
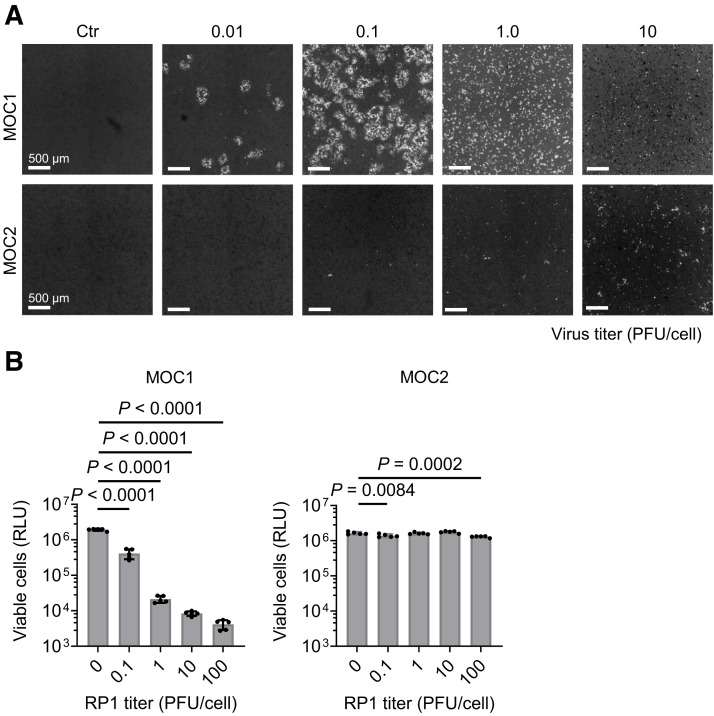
(A) Representative fluorescent images of MOC1 and MOC2 cells after 48 h of incubation with increasing titers of GFP-expressing RP1-15. GFP shown in gray scale. (B) MOC1 and MOC2 cell viability after 48 h with increasing RP1 titers in vitro, assessed via CellTiter-Glo assay. Significance determined using 1-way ANOVA with Dunnett test. RLU = relative light units.

To determine the infectious capacity of RP1, MOC1 and MOC2 cells were treated with increasing titers of the GFP-expressing RP1 and monitored by fluorescence imaging after 48 h. MOC1 cells showed dose-dependent GFP expression between 0.01 and 1.0 PFU/cell, whereas the highest tested titer (10 PFU/cell) led to low GFP expression because of reduced viability (Fig. 4A). In contrast, MOC2 cells exhibited signs of infection only with the highest titer (Fig. 4A). On the basis of RP1’s infection kinetics, the cytopathic behavior of RP1 was assessed across a broader range of virus titers (0.1–100 PFU/cell). After 48 h of incubation, MOC1 cell viability was significantly reduced compared with that of untreated cells (*P* < 0.0001) (Fig. 4B), whereas MOC2 cells showed minimal cytopathic effects except with the highest titer (Fig. 4B). Because of their high sensitivity to RP1, MOC1 cells were used for the in vivo experiments.

### Assessment of Systemic PD-L1 Response to Intratumoral RP1 Administration

**FIGURE 5.**
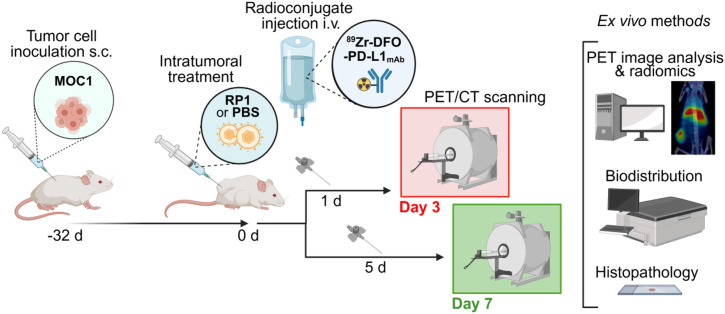
Experimental timeline of ^89^Zr-DFO-PD-L1_mAb_ immuno-PET studies to monitor PD-L1 expression after single intratumoral dose of RP1 or PBS in MOC1 tumor–bearing mice. Figure created with BioRender.com. i.v. = intravenous; s.c. = subcutaneous.

**FIGURE 6.**
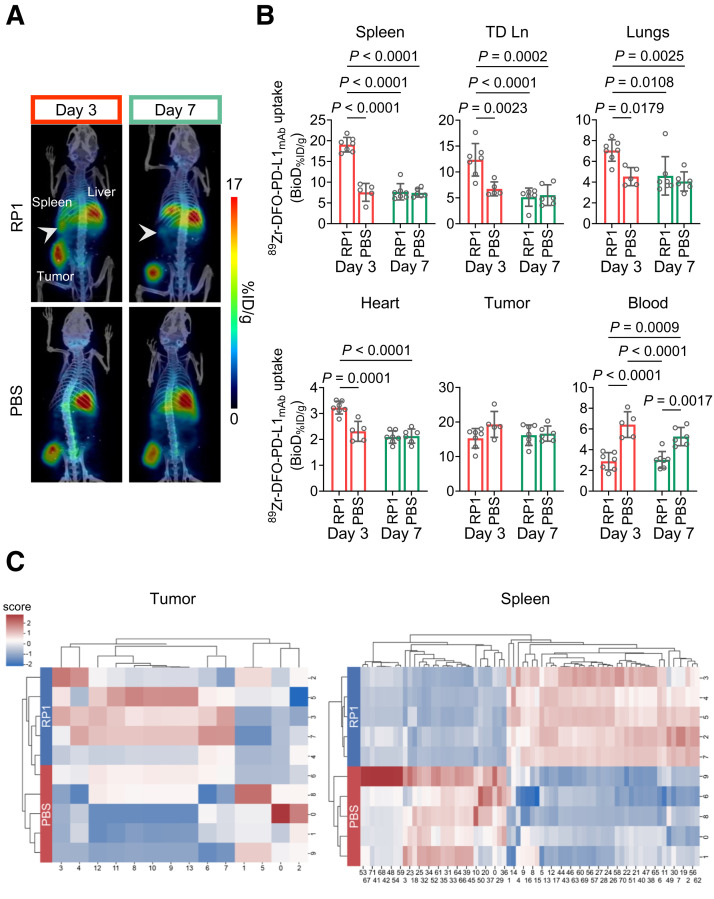
(A) Coregistered PET/CT scans of RP1 and PBS-treated mice on days 3 and 7 after treatment (PET, coronal slice; CT, 3-dimensional maximum intensity projection). Spleens of RP1-treated mice are highlighted (arrowheads). (B) Bar charts of ^89^Zr-DFO-PD-L1_mAb_ uptake on day 3 vs. day 7 after RP1 or PBS. Significance determined using 2-way ANOVA with Bonferroni adjustment on log-transformed data. (C) Radiomics heatmaps of tumor and spleen VOIs from day 3 scans. *x*-axis shows feature numbers from Supplemental Tables 3 and 4. Feature values normalized to *z* score. Decision trees shown to left of each heatmap. TD Ln = tumor-draining lymph node.

MOC1 tumor–bearing mice received a single intratumoral dose of the virus or vehicle (PBS), and responses were assessed 3 and 7 d after administration using immuno-PET with ^89^Zr-DFO-PD-L1_mAb_ (Fig. 5; *n* = 5–7 mice per group). The RP1 treatment was tolerated well, and minor reductions in tumor size were observed in the RP1 group compared with the control (Supplemental Fig. 5). On day 3, PET images and the biodistribution data showed significantly higher uptake of ^89^Zr-DFO-PD-L1_mAb_ in the spleen (*P* < 0.0001) and tumor-draining lymph nodes (*P* = 0.0023) in RP1-treated mice compared with controls (Figs. 6A and 6B; Supplemental Figs. 6A and 6B). These mice also showed significantly higher signals in the heart and lungs (Fig. 6B), suggesting a systemic impact of RP1 on PD-L1 expression. As a consequence, the increased uptake in these organs likely reduced radioconjugate levels in the blood of RP1-treated mice, resulting in lower systemic radioconjugate availability and tumor uptake (Supplemental Fig. 6B; Supplemental Table 2). By day 7, organ and tumor signals were similar between the groups (Fig. 6B; Supplemental Fig. 7; Supplemental Table 2), indicating that the effects observed on day 3 were transient.

Preliminary PET-based radiomics features in tumors and spleens of RP1- and PBS-treated mice 3 d after injection showed that, within the tumor volumes of interest, 14 features (1 shape-based, 5 first-order statistics-based, 8 texture-based) significantly differed between the treated and control groups (*P* < 0.05, Mann–Whitney *U* test) (Fig. 6C; Supplemental Table 3). First-order statistics-based features, derived from the global gray-level histogram of each volume of interest, include gray-level mean, maximum, minimum, kurtosis, and skewness. The first-order statistics-based features of kurtosis and skewness were significantly higher in RP1-treated tumors (*P* = 0.018 and 0.047, respectively; Supplemental Table 3), indicating greater deviation from normal distribution and increased intratumoral gray-level heterogeneity. Analysis of spleens revealed 72 significantly different features (including 5 shape-based, 16 first-order statistics-based, and 51 texture-based features) (Fig. 6C; Supplemental Table 4). Exploratory, within-dataset, cross-validated feature-based classifiers were built for both tumor and spleen radiomics features, performing with 80% and 100% accuracy, respectively (decision tree to the left of each heatmap in Fig. 6C).

**FIGURE 7.**
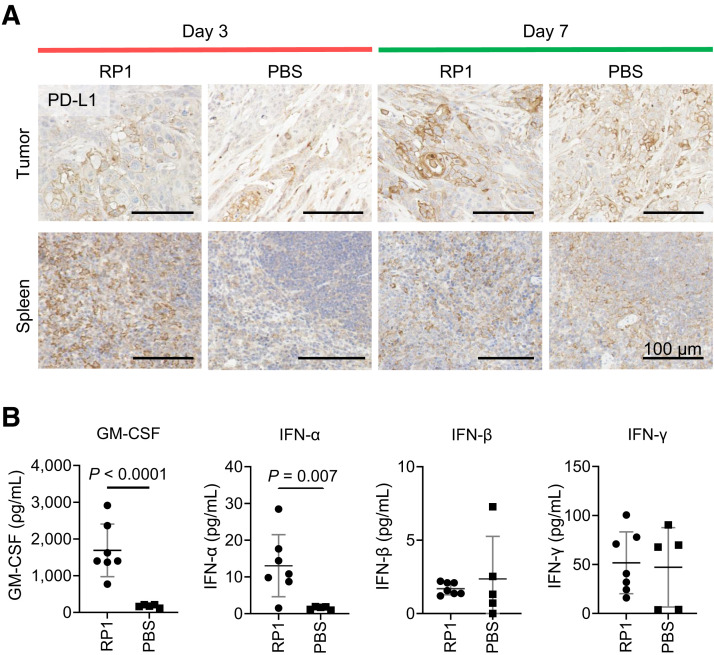
(A) PD-L1 immunohistochemistry of MOC1 tumor and spleen sections from immuno-PET studies on day 3 and 7 after single intratumoral RP1 or PBS dose. (B) Concentrations of GM-CSF, IFN-α, IFN-β and IFN-γ in tumors on day 3 after RP1 or PBS. Significance determined using multiple unpaired *t* tests on log-transformed data with Benjamini-Hochberg FDR correction (FDR < 1%).

Immunohistochemistry showed no clear difference in tumor PD-L1 expression between RP1- and PBS-treated groups on days 3 and 7 (Fig. 7A). However, the spleens of RP1-treated mice had markedly increased PD-L1 expression on day 3 (Fig. 7A). Furthermore, RP1-injected tumors showed significantly more tissue damage and increased GM-CSF levels, suggesting a direct oncolytic effect of the virus (Fig. 7B; Supplemental Figs. 7A–7C). Treatment with RP1 also led to a significant increase in tumor IFN-α on day 3, indicating an acute IFN response, whereas intratumoral IFN-β and IFN-γ levels remained similar between groups, possibly explaining the unchanged PD-L1 levels in PET studies (Fig. 7B). The increase of intratumoral CD8 T cells in RP1-treated tumors on day 7 further corroborated the immune stimulatory effect (Supplemental Fig. 7D).

## DISCUSSION

OVs are garnering increased interest as cytotoxic and immunomodulatory therapies against a range of cancers (7). Many studies have shown that OVs promote immune cell entry into the TME at the injection site. Until recently, their activity was thought to be restricted to local effects within injected lesions, and their potential to induce systemic responses in distant, noninjected (anamnestic) tumors remained debated (35). Immunomodulation of the TME is regarded as a key component of the therapeutic efficacy of OVs, contributing to intrinsic anticancer effects and the enhancement of ICIs. Various OVs have been shown to induce upregulation of PD-L1 expression within injected lesions, most likely because of IFN production after viral infection (9,36). However, data on OV-induced systemic immune modulation in uninjected tumors and secondary lymphoid organs (spleen, lymph nodes) remain limited because of challenges in tissue sampling.

Although originally developed as single-agent therapies, OVs show the greatest promise in overcoming resistance to ICI therapy. Recent clinical data for the OV RP1 (vusimogene oderparepvec) reported a 33% response rate with durable outcomes in 140 patients with melanoma whose disease had progressed on anti–PD-1 therapy. Nearly 50% had also received treatment with anti–cytotoxic T-lymphocyte associated protein 4 (37). In that study, paired tumor biopsies from responding and nonresponding patients revealed significant differences in inflammatory RNA-sequencing signatures. However, such complex tissue-based analyses are not feasible in routine clinical practice. Serial PD-L1 expression assessment on repeated biopsies, while indicative of favorable TME modulation, remains challenging outside of dedicated clinical trials and fails to capture dynamic treatment-related changes. As an alternative, immuno-PET imaging enables noninvasive, real-time monitoring of the spatiotemporal dynamics of PD-L1 expression, providing insight into the biologic effects of OVs and supporting the optimization of OV–ICI combination therapies.

In this context, PD-L1 immuno-PET is progressing toward clinical translation, with multiple tracers demonstrating acceptable safety, target engagement, and visualization of intrapatient heterogeneity across several cancers, including non–small cell lung cancer, head and neck cancer, and glioblastoma (32,38). In this study, we developed and characterized ^89^Zr-DFO-PD-L1_mAb_, an anti–PD-L1 antibody targeting the extracellular domain of murine PD-L1. After radiolabeling, immunoreactivity was preserved and the affinity for PD-L1 remained high, consistent with previously reported data (39,40). An in vivo dose-escalation study was used to establish the optimal injected antibody protein mass, with whole-body PET imaging and biodistribution analyses showing that higher coadministered doses of unlabeled PD-L1 mAb reduced splenic uptake of the radioconjugate.

Given the abundance of PD-L1-expressing immune cell populations, including dendritic cells and macrophages, in the spleen, it is essential to saturate this “antigen sink” with a sufficient amount of unlabeled antibody to improve radioconjugate availability at tumor sites (41). Additionally, FCγ receptor–expressing immune cells may further promote splenic uptake of full-length antibody conjugates (42). Using this optimized protocol, ^89^Zr-DFO-PD-L1_mAb_ provided proof of concept for detecting PD-L1 beyond primary tumor sites, as whole-body imaging enabled assessment of systemic immune organs such as spleen and lymph nodes. Clinically, such assessment is feasible, particularly with total-body PET allowing low-dose, dynamic, whole-organ quantification (43), although physiologic background in lymphoid tissues and the need for standardized analysis remain important considerations.

Interestingly, immuno-PET imaging showed that, compared with vehicle-treated controls, a single dose of the oHSV RP1 led to increased uptake of ^89^Zr-DFO-PD-L1_mAb_ in secondary lymphoid organs, including the spleen and tumor-draining lymph nodes, but not in MOC1 tumors on day 3. A similar increase in PD-L1 expression after local RP1 treatment was previously reported in a syngeneic melanoma model using ex vivo flow cytometry (36). As previous analysis confirmed that RP1 does not accumulate in nontumor tissues (data not shown), the observed PD-L1 upregulation in lymphatic tissues likely reflects elevated systemic cytokine levels. It may also result from increased trafficking of PD-L1–expressing dendritic cells carrying viral or tumor-associated antigens. These cells play a crucial role in tumor-draining lymph nodes during immunotherapy, as highlighted by Fransen et al. (44) and Dammeijer et al. (45), and are considered indicators of effective immune activation.

The present study demonstrates that immuno-PET provides a distinct advantage in capturing dynamic and systemic changes in PD-L1 expression, potentially supporting its use in guiding the timing of anti–PD-1 and anti–PD-L1 therapy after OV-induced immune activation. However, whereas immuno-PET enables assessment of net PD-L1 changes in vivo, it cannot resolve the contributing cellular populations within the TME, limiting direct comparisons between in vitro tumor cell expression and in vivo findings. In vivo, PD-L1 may be upregulated across multiple cell compartments and is strongly influenced by cytokine signaling within the TME.

Unlike conventional PET quantification of tumor volumes of interest, radiomics feature extraction with unsupervised clustering identified a distinct feature set that differentiated RP1-treated mice from control-treated mice on day 3 after injection. This underscores the ability of PET-based radiomics to capture intratumoral heterogeneity and tissue characteristics not detected by standard PET metrics. The added value of PET radiomics signatures over SUV-based readouts has also been demonstrated clinically in patients with B-cell lymphoma receiving immunotherapy, where a radiomics signature outperformed standard PET markers such as SUV_max_ and metabolic tumor volume (46). Our findings suggest that changes in tumor radiomics features reflect biologic responses induced by intratumoral RP1 injection, including elevated cytokines such as IFN-α, tumor necrosis factor-α, and GM-CSF. These cytokines promote immune cell infiltration and the formation of larger necrotic regions within tumors, thereby increasing intratumoral heterogeneity.

This is reflected by first-order histogram metrics, including higher kurtosis and altered skewness, consistent with more peaked intensity distributions driven by focal high-signal regions. Importantly, the radiomics changes observed in the spleen after treatment with RP1 are mechanistically consistent with a systemic immune activation cascade, triggered by oncolysis and innate immune sensing. Local release of damage-associated molecular patterns and type I IFNs activates tumor-resident antigen-presenting cells, which then migrate to secondary lymphoid organs to prime naïve T cells. This process is accompanied by transient splenic remodeling, including focal expansion of T cells in T cell–rich zones and transient PD-L1 expression on antigen-presenting cells and stromal elements. Such focal alterations in cellularity and checkpoint expression are expected to modify both first-order statistics-based and texture-based radiomics features, resulting in the higher kurtosis and altered skewness observed in RP1-treated spleens compared with controls.

Building on this foundation, future studies should incorporate rigorous coregistration of PET imaging with immunohistochemistry on excised specimens, increase cohort sizes to enable radiomics stratification of RP1-treated subgroups (including potential responder and nonresponder separation), and correlate spatial heterogeneity of PD-L1 expression with corresponding radiomics metrics to refine imaging biomarkers of treatment response. We recognize key limitations of the present work, including the evaluation of a single intratumoral injection and the measurement of PD-L1 levels at only 2 early postdosing time points, reflecting our focus on the early innate/type I IFN response. Accordingly, later time points and repeat-dosing regimens should be evaluated once feasibility and standardization have been established.

## CONCLUSION

These findings support the potential value of PD-L1-targeted immuno-PET to assess spatiotemporal changes in PD-L1 expression in the MOC1 model at whole-organism level to optimize OV therapy and guide the timing of PD-1/PD-L1 ICI initiation. PET radiomics analyses from primary lesions and secondary lymphoid organs may offer additional sensitivity to detect such changes, but they remain exploratory and require validation. Future preclinical studies across multiple tumor models with differing RP1 sensitivities, varied dosing regimens, and combinations with immune-checkpoint blockade will be necessary to determine whether ^89^Zr-DFO-PD-L1_mAb_ can robustly predict response to OV treatment.

## DISCLOSURE

This research was funded by the Oracle Cancer Trust, the Chellaram Foundation, and the ICR. Alan Melcher reports funding support from CRUK Programme Grant DRCRPGTD-Nov21\100001. Kevin Harrington reports funding support from CRUK Programme Grant DRCRPGTD-Nov21\100001 and Replimune. No other potential conflict of interest relevant to this article was reported.

## Notes

KEY POINTS **QUESTION:** Can PD-L1 immuno-PET detect the systemic effects of local OV in a murine model of head and neck cancer? **PERTINENT FINDINGS:** By applying PD-L1 immuno-PET after a single intratumoral injection of an oHSV in a syngeneic mouse model of HNSCC, we detected a transient increase of PD-L1 in systemic organs, including the spleen, but not locally in the tumor. Additional radiomics analysis provided insights into more subtle changes, detecting treatment-associated differences in spleens and injected tumors. **IMPLICATIONS FOR PATIENT CARE:** PD-L1 immuno-PET monitoring after OV may provide critical information on its systemic immune stimulatory effect, providing a platform to guide the onset of combinatorial treatments, including immune checkpoint blockade.

## References

[1] Johnson DE Burtness B Leemans CR Lui VWY Bauman JE Grandis JR. Head and neck squamous cell carcinoma. Nat Rev Dis Primers. 2020;6:92. 33243986 10.1038/s41572-020-00224-3 PMC7944998

[2] Topalian SL Drake CG Pardoll DM. Immune checkpoint blockade: a common denominator approach to cancer therapy. Cancer Cell. 2015;27:450–461. 25858804 10.1016/j.ccell.2015.03.001 PMC4400238

[3] Burtness B Harrington KJ Greil R.; KEYNOTE-048 Investigators. Pembrolizumab alone or with chemotherapy versus cetuximab with chemotherapy for recurrent or metastatic squamous cell carcinoma of the head and neck (KEYNOTE-048): a randomised, open-label, phase 3 study. Lancet. 2019;394:1915–1928. 31679945 10.1016/S0140-6736(19)32591-7

[4] Ferris RL Blumenschein G Jr Fayette J. Nivolumab for recurrent squamous-cell carcinoma of the head and neck. N Engl J Med. 2016;375:1856–1867. 27718784 10.1056/NEJMoa1602252 PMC5564292

[5] Elmusrati A Wang J Wang CY. Tumor microenvironment and immune evasion in head and neck squamous cell carcinoma. Int J Oral Sci. 2021;13:24. 34341329 10.1038/s41368-021-00131-7 PMC8329257

[6] Mandal R Şenbabaoğlu Y Desrichard A. The head and neck cancer immune landscape and its immunotherapeutic implications. JCI Insight. 2016;1:e89829. 27777979 10.1172/jci.insight.89829 PMC5070962

[7] Melcher A Harrington K Vile R. Oncolytic virotherapy as immunotherapy. Science. 2021;374:1325–1326. 34882456 10.1126/science.abk3436 PMC8961675

[8] Chiu M Armstrong EJL Jennings V. Combination therapy with oncolytic viruses and immune checkpoint inhibitors. Expert Opin Biol Ther. 2020;20:635–652. 32067509 10.1080/14712598.2020.1729351

[9] Samson A Scott KJ Taggart D. Intravenous delivery of oncolytic reovirus to brain tumor patients immunologically primes for subsequent checkpoint blockade. Sci Transl Med. 2018;10 10.1126/scitranslmed.aam7577 PMC6276984 29298869

[10] Liu Z Ravindranathan R Kalinski P Guo ZS Bartlett DL. Rational combination of oncolytic vaccinia virus and PD-L1 blockade works synergistically to enhance therapeutic efficacy. Nat Commun. 2017;8:14754. 28345650 10.1038/ncomms14754 PMC5378974

[11] Nakao S Arai Y Tasaki M. Intratumoral expression of IL-7 and IL-12 using an oncolytic virus increases systemic sensitivity to immune checkpoint blockade. Sci Transl Med. 2020;12:eaax7992. 31941828 10.1126/scitranslmed.aax7992

[12] Kelly KR Espitia CM Zhao W. Oncolytic reovirus sensitizes multiple myeloma cells to anti-PD-L1 therapy. Leukemia. 2018;32:230–233. 28832023 10.1038/leu.2017.272 PMC5844271

[13] Shen W Patnaik MM Ruiz A Russell SJ Peng KW. Immunovirotherapy with vesicular stomatitis virus and PD-L1 blockade enhances therapeutic outcome in murine acute myeloid leukemia. Blood. 2016;127:1449–1458. 26712908 10.1182/blood-2015-06-652503 PMC4797021

[14] Jiang H Rivera-Molina Y Gomez-Manzano C. Oncolytic adenovirus and tumor-targeting immune modulatory therapy improve autologous cancer vaccination. Cancer Res. 2017;77:3894–3907. 28566332 10.1158/0008-5472.CAN-17-0468 PMC5549681

[15] Mahalingam D Wilkinson GA Eng KH. Pembrolizumab in combination with the oncolytic virus pelareorep and chemotherapy in patients with advanced pancreatic adenocarcinoma: a phase Ib study. Clin Cancer Res. 2020;26:71–81. 31694832 10.1158/1078-0432.CCR-19-2078 PMC6942612

[16] Andtbacka RH Kaufman HL Collichio F. Talimogene laherparepvec improves durable response rate in patients with advanced melanoma. J Clin Oncol. 2015;33:2780–2788. 26014293 10.1200/JCO.2014.58.3377

[17] Thomas S Kuncheria L Roulstone V. Development of a new fusion-enhanced oncolytic immunotherapy platform based on herpes simplex virus type 1. J Immunother Cancer. 2019;7:214. 31399043 10.1186/s40425-019-0682-1 PMC6689178

[18] Sun C Mezzadra R Schumacher TN. Regulation and function of the PD-L1 checkpoint. Immunity. 2018;48:434–452. 29562194 10.1016/j.immuni.2018.03.014 PMC7116507

[19] Bensch F van der Veen EL Lub-de Hooge MN. Zr-89-atezolizumab imaging as a non-invasive approach to assess clinical response to PD-L1 blockade in cancer. Nat Med. 2018;24:1852–1858. 30478423 10.1038/s41591-018-0255-8

[20] Niemeijer AN Leung D Huisman MC. Whole body PD-1 and PD-L1 positron emission tomography in patients with non-small-cell lung cancer. Nat Commun. 2018;9:4664. 30405135 10.1038/s41467-018-07131-y PMC6220188

[21] Chatterjee S Lesniak WG Gabrielson M. A humanized antibody for imaging immune checkpoint ligand PD-L1 expression in tumors. Oncotarget. 2016;7:10215–10227. 26848870 10.18632/oncotarget.7143 PMC4891115

[22] Heskamp S Hobo W Molkenboer-Kuenen JDM. Noninvasive imaging of tumor PD-L1 expression using radiolabeled anti-PD-L1 antibodies. Cancer Res. 2015;75:2928–2936. 25977331 10.1158/0008-5472.CAN-14-3477

[23] Kurino T Matsuda R Terui A. Poor outcome with anti-programmed death-ligand 1 (PD-L1) antibody due to poor pharmacokinetic properties in PD-1/PD-L1 blockade-sensitive mouse models. J Immunother Cancer. 2020;8:e000400. 32041818 10.1136/jitc-2019-000400 PMC7057431

[24] Verhoeff S van de Donk PP Aarntzen EHJG. Zr-89-durvalumab PD-L1 PET in recurrent or metastatic (R/M) squamous cell carcinoma of the head and neck [abstract]. J Clin Oncol. 2020;38(suppl):3573.

[25] Boellaard R. Standards for PET image acquisition and quantitative data analysis. J Nucl Med. 2009;50(suppl 1):11S–20S. 19380405 10.2967/jnumed.108.057182

[26] Cheebsumon P Yaqub M van Velden FH Hoekstra OS Lammertsma AA Boellaard R. Impact of [^18^F]FDG PET imaging parameters on automatic tumour delineation: need for improved tumour delineation methodology. Eur J Nucl Med Mol Imaging. 2011;38:2136–2144. 21858528 10.1007/s00259-011-1899-5 PMC3228515

[27] Yushkevich PA Piven J Hazlett HC. User-guided 3D active contour segmentation of anatomical structures: significantly improved efficiency and reliability. Neuroimage. 2006;31:1116–1128. 16545965 10.1016/j.neuroimage.2006.01.015

[28] van Griethuysen JJM Fedorov A Parmar C. Computational radiomics system to decode the radiographic phenotype. Cancer Res. 2017;77:e104–e107. 29092951 10.1158/0008-5472.CAN-17-0339 PMC5672828

[29] Zwanenburg A Vallières M Abdalah MA. The image biomarker standardization initiative: standardized quantitative radiomics for high-throughput image-based phenotyping. Radiology. 2020;295:328–338. 32154773 10.1148/radiol.2020191145 PMC7193906

[30] Kramer-Marek G Kiesewetter DO Capala J. Changes in HER2 expression in breast cancer xenografts after therapy can be quantified using PET and ^18^F-labeled affibody molecules. J Nucl Med. 2009;50:1131–1139. 19525458 10.2967/jnumed.108.057695 PMC2787241

[31] Lindmo T Bunn PA. Determination of the true immunoreactive fraction of monoclonal-antibodies after radiolabeling. Methods Enzymol. 1986;121:678–691. 3523136 10.1016/0076-6879(86)21067-8

[32] Dar D Rodak M Da Pieve C. Imaging PD-L1 in the brain—journey from the lab to the clinic. Neuro Oncol. 2025;27:567–582. 39470381 10.1093/neuonc/noae190 PMC11812043

[33] Workman P Aboagye EO Balkwill F.; Committee of the National Cancer Research Institute. Guidelines for the welfare and use of animals in cancer research. Br J Cancer. 2010;102:1555–1577. 20502460 10.1038/sj.bjc.6605642 PMC2883160

[34] Abou DS Ku T Smith-Jones PM. In vivo biodistribution and accumulation of ^89^Zr in mice. Nucl Med Biol. 2011;38:675–681. 21718943 10.1016/j.nucmedbio.2010.12.011 PMC4527328

[35] Marabelle A Andtbacka R Harrington K. Starting the fight in the tumor: expert recommendations for the development of human intratumoral immunotherapy (HIT-IT). Ann Oncol. 2018;29:2163–2174. 30295695 10.1093/annonc/mdy423 PMC6290929

[36] Roulstone V Kyula J Thomas S. Immunomodulatory effects of a novel, enhanced potency gibbon ape leukaemia virus (GALV) fusogenic membrane glycoprotein-expressing herpes simplex virus platform with increased efficacy combined with anti PD-1 therapy [abstract]. Cancer Res. 2021;81(suppl):1917.

[37] Wong MK Milhem MM Sacco JJ. RP1 combined with nivolumab in advanced anti-PD-1-failed melanoma (IGNYTE). J Clin Oncol. 2025;43:3589–3599. 40627813 10.1200/JCO-25-01346 PMC12622257

[38] De Feo MS Pontico M Frantellizzi V Corica F De Cristofaro F De Vincentis G. Zr-PET imaging in humans: a systematic review. Clin Transl Imaging. 2022;10:23–36.

[39] Heskamp S Wierstra PJ Molkenboer-Kuenen JDM. PD-L1 microSPECT/CT imaging for longitudinal monitoring of PD-L1 expression in syngeneic and humanized mouse models for cancer. Cancer Immunol Res. 2019;7:150–161. 30459153 10.1158/2326-6066.CIR-18-0280

[40] Josefsson A Nedrow JR Park S. Imaging, biodistribution, and dosimetry of radionuclide-labeled PD-L1 antibody in an immunocompetent mouse model of breast cancer. Cancer Res. 2016;76:472–479. 26554829 10.1158/0008-5472.CAN-15-2141 PMC4715915

[41] Peng Q Qiu X Zhang Z. PD-L1 on dendritic cells attenuates T cell activation and regulates response to immune checkpoint blockade. Nat Commun. 2020;11:4835. 32973173 10.1038/s41467-020-18570-x PMC7518441

[42] Vivier D Sharma SK Zeglis BM. Understanding the in vivo fate of radioimmunoconjugates for nuclear imaging. J Labelled Comp Radiopharm. 2018;61:672–692. 29665104 10.1002/jlcr.3628 PMC6432633

[43] Sun YY Cheng ZP Qiu JF Lu WZ. Performance and application of the total-body PET/CT scanner: a literature review. EJNMMI Res. 2024;14:38. 38607510 10.1186/s13550-023-01059-1 PMC11014840

[44] Fransen MF Schoonderwoerd M Knopf P. Tumor-draining lymph nodes are pivotal in PD-1/PD-L1 checkpoint therapy. JCI Insight. 2018;3: e124507. 10.1172/jci.insight.124507 PMC6328025 30518694

[45] Dammeijer F van Gulijk M Mulder EE. The PD-1/PD-L1-checkpoint restrains T cell immunity in tumor-draining lymph nodes. Cancer Cell. 2020;38:685–700.e8. 33007259 10.1016/j.ccell.2020.09.001

[46] Ligero M Simó M Carpio C. PET-based radiomics signature can predict durable responses to CAR T-cell therapy in patients with large B-cell lymphoma. EJHaem. 2023;4:1081–1088. 38024636 10.1002/jha2.757 PMC10660117

